# Comparison of the clinical and radiographic appearance of the cervical vertebrae with histological and anatomical findings in an eight-month old warmblood stallion suffering from cervical vertebral stenotic myelopathy (CVSM)

**DOI:** 10.1186/s12917-019-2047-x

**Published:** 2019-08-15

**Authors:** Magdalena Szklarz, Anna Lipinska, Malwina Slowikowska, Artur Niedzwiedz, Krzysztof Marycz, Maciej Janeczek

**Affiliations:** 1 Department of Animal Physiology and Biostructure, Wroclaw University of Environmental and Life Sciences Faculty of Veterinary Medicine, ul Kozuchowska 1/3, 51-631 Wroclaw, Wrocław, Poland; 2 Department of Internal Diseases with Clinic for Horses, Dogs and Cats, Wroclaw Univeristy of Environmental and Life Sciences, Faculty of Veterinary Medicine, Pl. Grunwaldzki 47, 50-366 Wroclaw, Wrocław, Poland; 3 Department of Experimental Biology, Wroclaw University of Environmental and Life Sciences, Faculty of Biology and Animal Science, ul Norwida 27B, 50-375 Wrocław, Poland

**Keywords:** Spinal cord compression, Cervical vertebral stenosis, Ataxia, Horses

## Abstract

**Background:**

Cervical vertebral stenotic myelopathy (CVSM) remains one of the most important abnormalities of the cervical spine resulting in neurological deficits in horses. The aim of the following study was to compare the results of the clinical and neurological examination, the results of myelography and the post mortem anatomical and histological appearance of the spinal cord and cervical vertebrae in a horse with CVSM.

**Case presentation:**

The following study describes a clinical case of an eight-month-old stallion with ataxia. Plain cervical radiographs indicated narrowing of the spinal canal. Conservative therapy using NSAIDs did not result in any improvement in the gait of the horse. Due to economic constraints, surgical intervention was excluded. The owner chose to humanely euthanise the horse. Immediately after euthanasia, post mortem myelography was performed, and measurements of the myelographic dye column were taken. They revealed a 67% DMC reduction and a 64% DD reduction at the C3/C4 level. Afterwards, an anatomical dissection was performed. The cervical vertebrae and vertebral canal were macroscopically inspected and measured and indicated a 44% narrowing of the canal at the C3/C4 level. The spinal cord was removed and underwent histological evaluation after staining. Microscopic lesions were visible at the level of the compression and included axonal degeneration with partial or complete loss of myelin in the white matter of the lateral and dorsal funiculi as well as the formation of dysfunctional so-called “spongy structures”. An increase in the number of microglial cells and collagen was also observed. The formation of glial scars was excluded. Immunohistochemical studies revealed a negative transmembrane glycoprotein CD68(−) – monocyte response and a negative tumor necrosis alpha TNFα (−) reaction.

**Conclusions:**

CVSM may be difficult to diagnose, even for experienced veterinary surgeons. Currently, an ex vivo histopathologic examination of the spinal cord is thought to be the gold standard in the diagnosis of CVSM. Our histological examination revealed no CVSM-specific glial scar formation and a CD68(−) negative and TNF-α negative reaction, which have not been previously reported. Histological lesions in CVSM may vary depending show inter-individual variability and on the treatment, which further hinders ex-vivo diagnostics.

## Background

Cervical vertebral stenotic myelopathy (CVSM), also known as wobbler syndrome or cervical vertebral malformation or malarticulation (CVM) can affect both young and old equine patients [5, 17, 18, 21, 24]. The disease is divided into a dynamic (type I) or static (type II) type, depending on the type, location and nature of the lesions as well as the age of the patient [27, 31, 37]. Clinical symptoms can vary from slight hypermetria, through lack of balance, neck and back stiffness, incoordination to spontaneous falling. As horses continue to be important companion and sport animals, their owners have become more aware of their health. Equine ataxia affects a relatively large number of horses. However, there are limited diagnostic methods that allow its correct identification [6, 20, 22, 38]. The prognosis associated with ataxia is usually poor, and the costs of treatment of horses with ataxia, including surgical intervention, are usually very high [10, 26, 34]. The correct diagnosis of CVSM in horses is particularly important, given that there are only a few facilities in Europe that are able to manage the disorder surgically [34, 39]. The aim of the study was to present the results of the clinical, orthopedic, neurological, radiological examinations of a young horse suffering from CVSM. Due to poor prognosis, the horse was euthanized and tissue sections were collected *post-mortem*, enabling a histological assessment of the spinal cord. This prompted us to assess the correlation between symptoms of ataxia and the histological lesions of the spinal cord. The obtained results differed from available data and encourage further investigation of equine CVSM, considering that spinal cord histological studies were considered the ex-vivo gold standard of CVSM diagnosis in horses.

## Case presentation

### Case details

An eight-month-old warmblood stallion was presented for examination due to ataxia manifested by problems with coordination, stumbling and spontaneous falling. The symptoms were first observed at the pasture when the horse grazed with other colts. There were no direct signs of any trauma at pasture. There was no history of ataxic horse in the family, the horse was raised in one location and was not transported. It did not have contact with unknown horses/ animals or sport horses.

### Clinical and diagnostic findings

The horse underwent a clinical examination consisting of observation, palpation and auscultation. The horse did not present any signs of pain. No nasal discharge or coughing, oedema or wounds/scars has been detected. Urination, defecation and feed uptake were normal. Body temperature measurement, heart and lung auscultation including respiratory and heart rates were in physiological values. Mucous membranes were pink with CRT < 2 s. Palpation of the lymph nodes did not reveal any abnormalities. Both testes has been palpable in the scrotum. Ophthalmologic examination showed no abnormalities. There were no other clinical signs of infectious diseases. Laboratory blood analysis, which included a complete blood count and serum chemistry as well as micro- and macro-element and vitamin E analysis was performed and did not reveal any abnormalities. The orthopedic examination revealed gait deficits, graded as 3–4/5 according to the Mayhew scale [21, 23]. The neurological examination revealed no changes in the mental status of the horse. There were no cranial nerve deficits and all spinal reflexes were normal [14, 20]. The patient presented hypermetria during walk and trot. While resting in the box, the horse tried to find a stable, comfortable position and moved only when necessary. The horse also had problems with turning and kept its inner hind leg on the ground. Additionally, the horse could not be walked backward there was risk of loss of balance and falling. Based on the neurological examination, the lesion was localised to the cervical spinal cord [14, 20]. It was therefore decided to perform standing lateral radiographs of the cervical vertebrae, which did not reveal any malformation or malallineation changes. The differential diagnoses included CVSM, aberrant parasite migration and equine degenerative myeloencephalopathy (EDM). Equine protozoal meningitis (EPM), due to geographic location, was excluded [7, 11, 28]. The radiographs were evaluated for the measurements of the cervical inter- and intravertebral ratio (Table. 1). The lowest sagittal intravertebral ratio was noted at the C3/C4 level and amounted to 31.69% with 55.06% sagittal intervertebral ratio, while the values obtained at the C4/C5 level amounted to 47.77 and 50.54%, respectively. It was decided not to perform a CSF tap following an analysis of the radiograms.

**Table 1:** Measurements of inter-, intravertebral ratio, DMC and DD reduction according to plain radiographs and the myelogram

Localisation	Sagittal intervertebral ratio	Sagittal intravertebral ratio	Dorsal Myelographic Column	Dural Diameter
C1/C2	82.63%	C2 73.36%	–	90.95%
C2/C3	66.28%	C3 50.93%	85.98%	78.84%
C3/C4	55.06%	C4 31.69	33% (67% red.)	36% (64% red.)
C4/C5	50.54%	C5 47.77%	67.16%	81.22%
C5/C6	49.95%	C6 51.27%	90%	78.82%
C6/C7	60.21%	C7 49.95%	61.79%	79.05%

Due to financial constraints, conservative therapy was attempted, and included an administration of nonsteroidal anti-inflammatory drugs (flunixin meglumin 1.1 mg/kg BW q24h),^1^ a high dose of vitamin E^2^ (10,000 IU/day) and exercise restriction. A control examination performed after a month of treatment did not reveal any improvement in the horse’s clinical findings. At that stage, it was clear that the horse would not be fit for sport use and the owner did not agree to further examinations. Due to the high degree of ataxia of the horse, which posed a threat to the animal itself as well as its caregivers and no possibility of effective treatment, the animal was euthanised. General anaesthesia was induced by diazepam^3^ (0.02 mg/kg IV) and ketamine^4^ (2.2 mg/kg IV), after achieving sufficient sedation with xylazine^5^ (1.1 mg/kg IV), and euthanasia performed by administration of pentobarbital sodium^6^ (140 mg/kg IV after receiving sufficient anaesthesia).

### Post-mortem evaluation

**Fig. 1:**
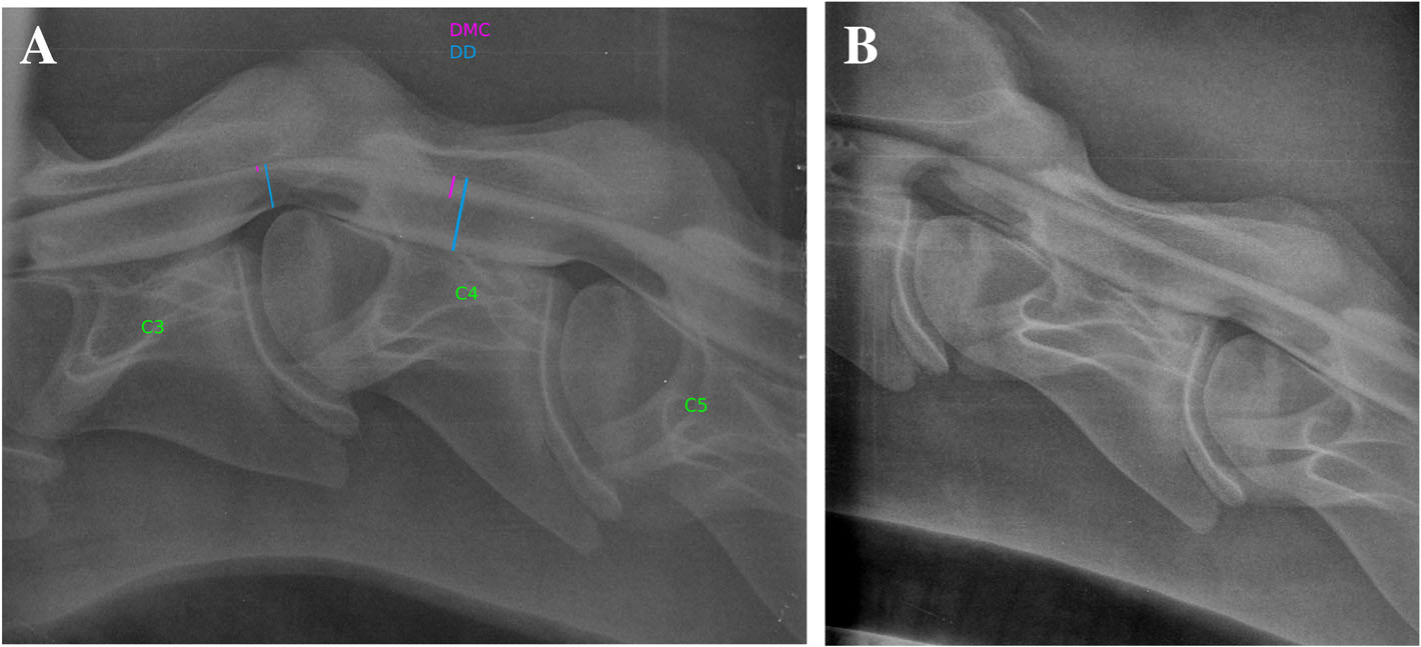
A- Lateral myelogram projection presenting the C3-C5 cervical vertebrae in a flexed position showing a significant reduction of the dorsal dye column at the level of the C3/C4 articulation indicating spinal canal narrowing and suggesting compression of the spinal cord. The reduction of the ventral dye column is typical for cervical flexion radiographs. The dural diameter and dorsal myelographic column measurements are marked. B- Lateral myelogram projection presenting the C2-C4 cervical vertebrae in a neutral position of the neck. No degenerative joint disease or malformation changes were noticed at the level of C3/C4

**Fig. 2:**
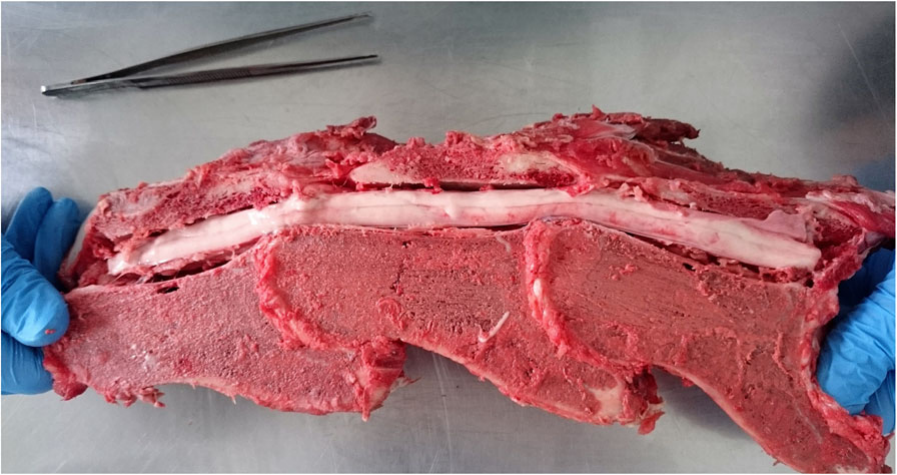
Anatomical dissection with longitudinal cross section of the cervical spine presenting the C3-C5 region

Immediately after euthanasia and after obtaining the owner consent, myelography was performed to confirm the diagnosis. The horse was placed in right lateral recumbency. A non-ionic contrast medium (*Accupaque® 350*^7^*;* 10 ml/100 kg iohexol 350 mg I/ml^e^) was slowly injected subarachnoidally (Spine- Ject®, 18G, 3 1/2″) into the atlanto-occipital space after removing an equal quantity of cerebrospinal fluid. The diagnostic procedure consisted of radiographs in neutral, flexed, and extended positions of the neck. The dorsal myelographic column (DMC) and the dural diameter (DD) were measured based on the myelographic dye column. The measurements of the myelographic dye column revealed a 67% reduction of the dorsal myelographic column (DMC) and a 64% decrease in the dural diameter (DD) at the level of C3/C4 (Fig.1A, B, Tab.1) in a flexed spine position, which confirmed type I CVSM (a dynamic lesion). Next, an anatomical dissection was performed. The cervical vertebral column was removed and immersed in a tank of neutral buffered formalin for 1 week, than sectioned saggitally [33]. The cervical vertebrae and the vertebral canal were macroscopically inspected to obtain actual measurements at the stenotic site. Real scenario measurements of the vertebral canal were taken during the anatomical dissection and are summarised in Table 2 (Fig. 2). According to the measurements, there was a 44% narrowing of the vertebral canal at C3/C4 level and 27% narrowing of the vertebral canal at C4/C5 level respectively. The spinal cord was collected as fast as possible after euthanasia. Following fixation, the segments of the C3/C4 spinal cord compression were sectioned transversely and divided into several parts.

**Table 2:** Real scenario measurements of vertebral canal taken at the level from C3 to C5

Localisation	Measurement in mm
C3	18
C3/4	10
C4	18
C4/5	13
C5	19

### Histopathologic and morphologic examination

All the samples were fixed in 4% buffered formalin and embedded in paraffin blocks. Five μm paraffin sections were obtained using a rotary microtome, and they were stained with hematoxylin and eosin (HE) and Masson trichrome (MTC) according to the relevant histological protocols. Section analysis was carried out with an optical microscope (Axio Imager A1; Carl Zeiss).

### Immunohistochemistry

#### CD68/KP1

For immunohistochemistry analysis, the paraffin embedded tissue was cut into 4 μm thick section, placed on silanized slides (Dako, S 3003) and dried for 12 h in an incubator at 37 °C. The dried sections were then dehydrated and they were pre-treated with 0.01 M citrate buffer solution at a higher pH in an incubator at 97 °C for 20 min to unmask the antigenic sites. The sections were rinsed in 0.01% phosphate buffered saline (PBS). Then, they were treated with enzyme block FLEX peroxidase for 5 min, rinsed in PBS and subsequently overlaid by the primary antibody CD68/KP1 (Dako). Next, the slides were rinsed in PBS, labelled using a Polymer Flex/HRP for 20 min, rinsed in PBS for 5 min and covered by Chromogen Flex DAB with Subchromogen for 10 min. After rinsing the slides in PBS, they were counterstained using FLEX Hematoxylin for 5 min, washed with deionized water and PBS and analysed with an optical microscope (Zeiss Axio Scope A1; Carl Zeiss). A positive and a negative control were performed for each sample.

#### TNF-α

Immunostaining was performed using a standard technique, according to protocols designed at the Department of Experimental Biology, Faculty of Biology and Animal Science at the Wroclaw University of Environmental and Life Sciences [2]. The tissue samples were cut into 3 μm-thick sections, deparaffinised in xylene and washed in a series of decreasing alcohol concentrations from 100 to 50%. An EnVision System (Dako) was used to visualize the antigen-antibody reaction. Immunoperoxidase labelling was performed using polyclonal antibodies against TNF-α (R&D Systems, USA). Antigen heat-induced retrieval was performed by incubating the slides with a target retrieval solution (pH 9.0; Dako) for 20 min at 96 °C. The endogenous peroxidase activity was blocked with 3% hydrogen peroxide, and the tissue sections were then washed with Tris-buffered saline (TBS) for 5 min at room temperature. Next, the slides were labelled with primary antibodies for 20 min at 20 °C. The antibodies were diluted to 1:10. Then the sections were counterstained with Mayer’s hematoxylin for 1 min, washed with tap water, and rehydrated in increasing ethanol concentrations from 50 to 100%, closed in a mounting medium with coverslips and analysed using an optical microscope (Axio Imager A1; Carl Zeiss).

**Fig. 3:**
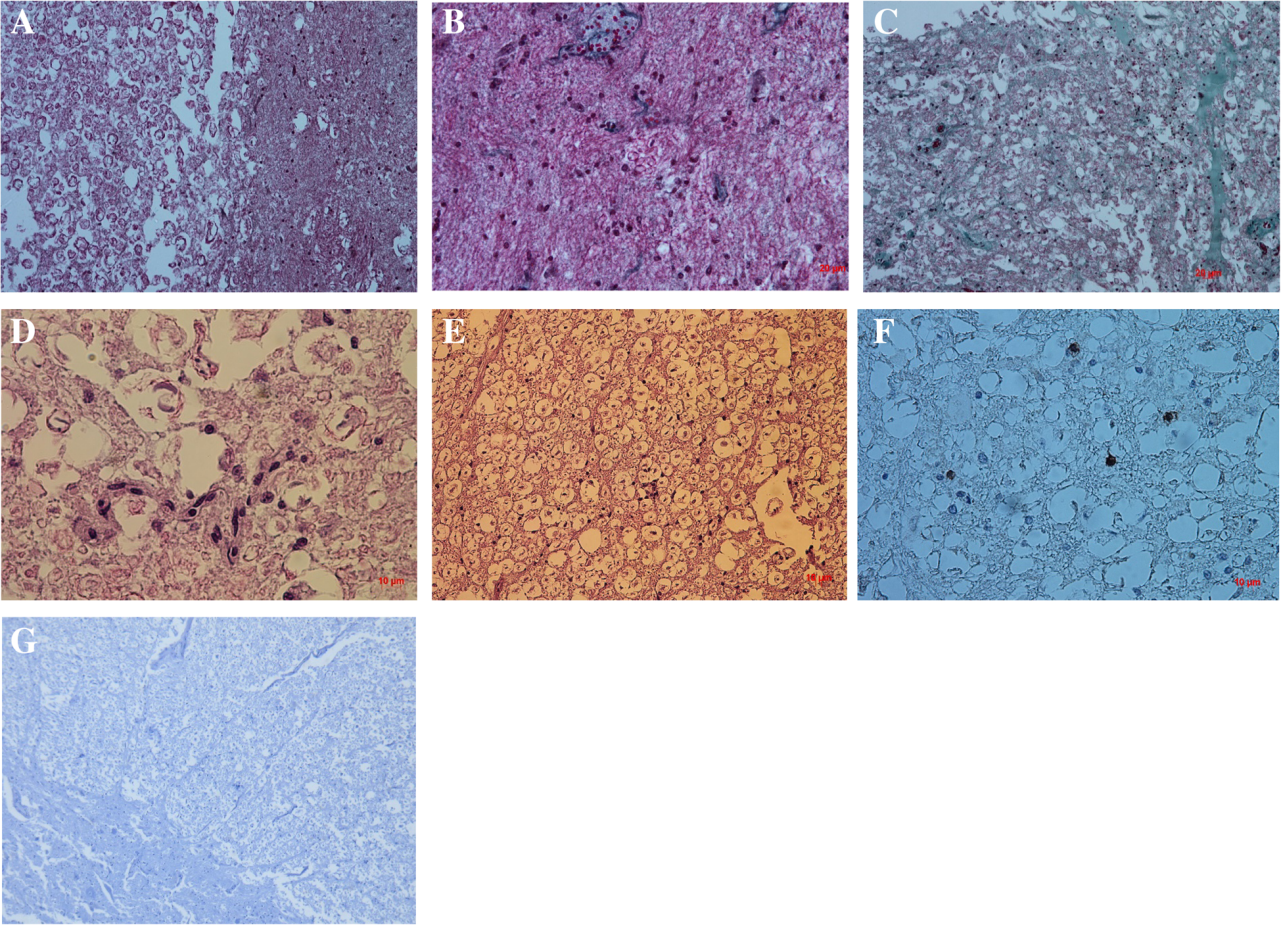
Histological and immunohistochemical examination of equine spinal cord compression stained with hematoxylin and eosin (H&E) (**a**, **d**, **e**) and Masson’s trichrome staining (**b**, **c**): A- The difference between the white and grey matter at the site of spinal cord compression. **b**, C-The changes associated with an increased number of microglial cell infiltration and perivascular collagen D-Multiple digestive chambers forming a spongy structure with a macrophage infiltration (**f**); F- Expression of CD68 with few visible /several macrophages; G- Expression of the tumor necrosis factor –alpha (TNF-α)

The histological studies at the level of spinal cord compression revealed an axonal degeneration with partial or total loss of myelin (Fig.3A) as well as an increase in the number of microglia cells (Fig.3B), which later transformed into tissue macrophages responsible for phagocytosis and removal of debris from the damaged myelin sheaths. Macrophages loaded with phagocytized material (so-called “gitter cells”) were observed in the form of clusters of cells along blood vessels and around the “digestive chamber” (Fig.3B, Ds). Numerous “digestive chambers” (myelin sheath fragments being phagocytosed by macrophages**)** formed in the areas of the **s**pinal cord that did not contain myelin, forming dysfunctional areas with a spongy structure typical for the wobbler syndrome (Fig.3E). The described changes generally affected the white matter- the lateral and dorsal funiculi, which are more sensitive to pressure or compression and are the first to undergo changes. In this horse, the axonal degeneration consisted of partial or complete loss of myelin sheaths or their transformation into swollen or spherical structures (Fig.3E). Moreover, the increase in perivascular collagen (Fig. 3B, C) was observed directly at the level of the spinal cord compression, whereas no gliosis (so-called astrocytic scar) was spotted, which may indicate an initial stage of the disease or its occurrence far from the place of compression. Immunohistochemical studies showed a negative transmembrane glycoprotein CD68 (−) monocyte response, by circulating and tissue macrophages such as microglia (Fig.3F) and a negative tumor necrosis factor alpha (TNF-α (−)) reaction (Fig.3G) – a.k.a. X.

## Discussion and conclusions

This case describes a young male horse presenting severe gait deficits. Cervical vertebral stenotic myelopathy (CVSM), aberrant parasite migration and equine degenerative myeloencephalopathy (EDM) were considered in the differential diagnostics. Due to the geographic location, equine protozoal meningitis (EPM) was excluded. The histological lesions and the results of the examinations excluded other diseases, such as EDM or a nematode infection. EDM affects animals that are genetically predisposed to the disease, that have disorders of vitamin E absorption, limited access to pasture or excess exposure to insecticides, all of which were excluded in the presented case [3, 25]. The histological lesions visible in the course of EDM affect the dorsal grey column, while those in CVSM affect the white matter. EDM features neuroaxonal dystrophy with axonal-dendritic edema and neuronal atrophy, which results from lipofuscin accumulation. The histological findings in the case of chronic, eosinophilic encephalomyelitis caused by nematodes from the *Protostrongylidae* family are characteristic [1]. Most migration routes are present in the white matter or on the border between the grey and white matter and lead to hemorrhagic lesions, large eosinophilic infiltrations and infiltrations of multinucleated giant cells, lymphocytes and hemosiderin-laden macrophages. Adult parasites are found mainly in the brain, while larvae are present in the thoracic and lumbar spinal cord and are located mainly in the dorsal grey columns [35]. Cellular vacuolization and axonal spheroids indicate cellular lesions and occur in various neurological diseases. Parasytoses caused by cerebrospinal nematodes such as *Parelaphostrongylus tenuis* lead to dorsal grey column and lateral white column lesions, but are not reported in our geographical region. Our results indicated a significant reduction in the functionality of the spinal cord nerve tissue following loss of neurons which is similar to some other diseases. At the same time the lack of haemorrhagic lesions, lipofuscin deposits or hemosiderin-laden cells excludes those diseases. The presence of neuronal degeneration, numerous microglial cells and unmyelinated dysfunctional so-called “spongy structure” areas all suggest CVSM, which was confirmed in the radiological findings. Most studies reporting age and breed correlations of CVSM describe the condition in young fast-growing males [15, 18, 19, 26, 27, 34, 39]. The latest research indicates that the traditional 50% reduction of DMC [8] rule is ineffective in diagnosing CVSM, particularly in the flexed neck position [27, 31, 34, 37]. Other studies report the principle of diagnosis based on comparing the intra- and intervertebral ratio measured on cervical spine lateral radiographic projections. However, such measurements are limited due to observer bias. Nevertheless, static and dynamic CVSM are distinguished [27, 34, 37]. A 70% DMC reduction instead of a 50% one has already been proposed to avoid false positive results [22, 37, 38]. Therefore, radiographs and myelograms should be evaluated with caution and always in correlation with the results of the clinical examinations. Moreover, the lateral view is useful only in detecting dorso-ventral compression [31, 38]. In the described stallion, the compression, understood as a reduction of the DMC equal or higher than 50%, was only detected at the C3/C4 level. The measured DMC reduction in this colt was 67%, which is thought to be responsible for the clinical symptoms. A 20% DD reduction has been described as potentially significant enough to reflect dural sac narrowing due to vertebral canal malformation [27, 37, 38]. In the presented study, the obtained DD reduction was 64% at the C3/C4 level. Contrary to hypothetic results based on the radiographs, the post-mortem measurements revealed a 44% diameter reduction of the vertebral canal. These measurements were taken in a neutral position of the spine. It is clear that the measurements taken post mortem are more accurate than the radiographic analysis as they were collected in a neutral position of the cervical spine following confirmation of dynamic changes. Interestingly, the percentage reduction was lower than CVSM gold standard value, while the clinical symptoms were described as moderate to strong. Hence we expected a higher reduction rate based on measurements performed on anatomical sections. The intra- and intervertebral ratio in the C4/C5 section should be taken into account with reference to the available literature [9]. We focused on the C3/C4 compression due to the lowest intravertebral ratio at this site with a simultaneous reduction of the DD and DMC as well as measurements carried out macroscopically. Despite the fact that the value of the intravertebral ratio at the C4/C5 site was lower than 48.5%, and that the intervertebral ratio considered more specific was lower than at C3/C4, which, based on the cited study, could have high diagnostic value, was not confirmed in the measurements performed during the myelography [8, 9]. This may have been caused by differences in the assessment of the cervical radiographs [12]. However, myelography is considered an important intravital diagnostic tool that enables the qualification of patients for cervical vertebral stabilisation surgery, therefore our study focused on the C3/C4 analysis. CSF analysis which is also a valuable tool concerning protozoal or verminous infection has not been performed due to its high costs and low diagnostic value in the case of suspected CVSM [29]. CT and MR are also valuable tools that enable the analysis of the cervical spine. Due to technical constraints, MR can be currently used to assess only the cranial section of the cervical spine and enables high quality soft tissue analysis of the studied area. Computed tomography and myelo-CT are valuable diagnostic methods but require an appropriate gantry size [16]. Unfortunately, high-field imaging of the cervical spine using MR or appropriate CT is not available in Poland. We decided to carry out a histologic study, which is thought to be the most appropriate tool in the diagnosis of CVSM [27, 37] although it is not applicable in in vivo studies. The typical histologic changes described in horses with CVSM are observed within the white matter [42]. Commonly described primary changes consist of an increased number of macrophages, perivascular collagen [41], the presence of “digestive chambers” [36, 40] partial/total loss of myelin, axonal degeneration [13, 40, 41] and gliosis [41, 42]. Our results were slightly different as they did not include some of the commonly described histologic changes such as the formation of a glial scar or spheroids characteristic of CVSM (only oedematose axons were found). This may result from an early NSAIDs therapy, which inhibited the inflammatory process, the age of the animal or the relatively short interval between symptom recognition and euthanasia. The results of the immunohistochemical study, which was not performed by other authors, did not confirm inflammation. In the case of CD68, the lack of reaction may indicate non-specificity of the used antibody [4] or the occurrence of a very weak reaction at the limit of detection [30], which is difficult to interpret. However, the negative TNFα reaction indicates an absence of the tumor necrosis factor representative of inflammation, produced by the activated tissue macrophages [32, 36]. As a result of the administration of anti-inflammatory drugs, the inflammatory reaction was suppressed or the histologic changes did not develop sufficiently due to a rapid diagnosis of the disease as evidenced by the lack of gliosis. Nevertheless, it would be useful to collect a sample from the spinal cord both above and below the place of the compression while performing a histologic examination. However, a complete histological and immunohistochemical analysis of the entire cervical spinal cord is very difficult technically. An analysis of spinal cord samples above and below the described lesion may have been performed to confirm or exclude co-existing diseases. In addition, a wider sample analysis may have facilitated the identification of an area with lesions typical of CVSM and an analysis of their severity depending on their distance from the site of greatest compression.

CVSM is a common, non-infectious equine disease that is caused by narrowing of the cervical spinal canal and myelocompression, leading to ataxia. Despite numerous studies on CVSM, it is still unclear how the risk of the disease can be minimised. Effective treatment is time-consuming, cost-intensive and difficult, reducing the chance of a full recovery. The diagnosis of CVSM in the equine patient remains challenging. Apart from a thorough orthopaedic and neurologic examination, plain radiographs and myelograms remain the only available tool for in vivo CVSM diagnostics. Even though histopathology is currently thought to be the most appropriate tool in CVSM diagnosis, it can only be carried out *post-mortem*. Our findings suggest the histological lesions in CVSM may differ between individuals and depend on the type of treatment, which limits ex-vivo diagnosis of the disease. In the described case, the histological analysis excluded the presence of a glial scar specific for CVSM. In addition, CD68(−) negative and TNF-α negative reactions were detected, which have not been reported previously. Furthermore, most CT or MRI devices are not available for cervical vertebral examinations due to size constraints. Hence, if only possible, in our opinion routine *post-mortem* histological and immunohistochemical analyses should be performed in horses euthanized due to CVSM for further examination of the disease.

## Data Availability

Most of the data generated or analysed during this study are included in this published article and its supplementary information files. All data are available from the corresponding author on reasonable request.

## Abbreviations

CVM: Cervical Vertebral Malformation
CVSM: Cervical Vertebral Stenotic Myelopathy
DD: Dural Diameter
DMC: Dorsal Myelographic Column
HE: Hematoxylin and Eosin
MTC: Masson Trichrome
NSAIDs: Non-Steroidal Anti-Inflammatory Drugs

## Footnotes

1
*Vet Agro, Lublin, Poland*

2
*Vetoquinol Biowet Sp. z o.o., Gorzow Wielkopolski, Poland*

3
*Warszawskie Zakłady Farmaceutyczne POLFA S.A., Warszawa, Poland*

4
*Vetoquinol Biowet Sp. z o.o., Gorzow Wielkopolski, Poland*

5
*Vet Agro, Lublin, Poland*

6
*Veterinary Pharmaceutical Industry, Ozzano Emilia, Italy*

7
*GE Healthcare, Little Chalfont, UK*
